# Learning Curves for Laparoscopic Repair of Inguinal Hernia and Communicating Hydrocele in Children

**DOI:** 10.3389/fped.2017.00207

**Published:** 2017-09-27

**Authors:** Catarina Barroso, Péter Etlinger, Ana Luísa Alves, Angélica Osório, José Luís Carvalho, Ruben Lamas-Pinheiro, Jorge Correia-Pinto

**Affiliations:** ^1^Department of Pediatric Surgery, Hospital Braga, Braga, Portugal; ^2^School of Medicine, Life and Health Sciences Research Institute (ICVS), University of Minho, Braga, Portugal; ^3^ICVS/3B’s Associate Laboratory, Braga, Portugal

**Keywords:** inguinal hernia, communicating hydrocele, children, laparoscopy, percutaneous internal ring suturing, learning curve

## Abstract

**Introduction:**

We analyzed the department and surgeon learning curves during implementation of the percutaneous internal ring suturing (PIRS) technique in our department.

**Methods:**

Children proposed for inguinal hernia or communicating hydrocele repair were included (*n* = 607). After mentorship, all surgeons were free to propose open or PIRS repair. From gathered data, we assessed department and surgeon learning curves through cumulative experience focusing in perioperative complications, conversion, ipsilateral recurrence, postoperative complications, and metachronous hernia, with benchmarks defined by open repair.

**Results:**

Department-centered analysis revealed that perioperative complications, conversion, and ipsilateral recurrence rates were higher in the beginning, reaching the benchmarks when each surgeon performed, at least, 35 laparoscopic repairs. Postoperative complications and metachronous hernia rates were independent from learning curves, with the metachronous hernia rate being significantly lower in PIRS patients. During the program, the percentage of males in those operated by PIRS progressively increased reaching the percentage of males, in our sample, when department operated over 230 cases.

**Conclusion:**

Thirty-five laparoscopic cases per surgeon are required for perioperative complications, conversion, and ipsilateral recurrence reach the benchmark. The gap between the percentage of males, in those operated by PIRS and in those proposed for surgery, monitors the confidence of the team in the program.

## Introduction

For years and years, infants and children with surgical indication for repairing inguinal hernia or communicating hydrocele were treated with high ligation and division of the sac by an open inguinal approach. Around the 1990s, after the first report of a laparoscopic inguinal hernia repair (1), several techniques have been described that can be clustered in two major groups: intracorporeal techniques that generally comprise dissection, ligation, and division of the sac similarly to the classic inguinal approach (true herniotomy) (2–5) and extracorporeal percutaneous techniques that just ligate the patent *processus vaginalis* without division (6–15). Even though no consensus exists favoring any of the techniques, there are enough evidence-based data supporting minimally invasive repair as a safe and effective method, if proper training and mentorship are assured (16).

A few years ago, our department decided to implement a minimally invasive program to repair inguinal hernia and communicating hydrocele embroiling all staff members. After a systematic review and mentorship, the percutaneous internal ring suturing (PIRS) technique (11) leaving no peritoneal gaps was selected and implemented. It favored our choice, the satisfactory cosmesis and the possibility to identify the patency of the contralateral *processus vaginalis* (17); among potential disadvantages, we had some reports mentioning higher rates of complications and recurrence (18, 19).

Herein, we evaluate our department- and surgeon-centered learning curves trying to extract some lessons we can share with other centers implementing a similar program.

## Materials and Methods

### Population and Data Collection

This study was approved by the scientific ethic committee from our institution with the reference: SECVS 133/2014. All staff members involved in the program were consultants with basic training as pediatric surgeons and different skill levels in laparoscopic surgery. We included all children submitted to surgical repair of indirect inguinal hernia (at any age) or communicating hydrocele (older than 2 years old) since June 2011 until November 2016 in our department. The patients were either operated by open approach (OA group) or by percutaneous internal ring suturing (PIRS group). The decision of proposing the minimally invasive approach was surgeon-dependent, and determined by each surgeon’s experience, beliefs, and confidence on the technique. Patients with hernias other than indirect inguinal hernia were excluded.

**Figure 1:**
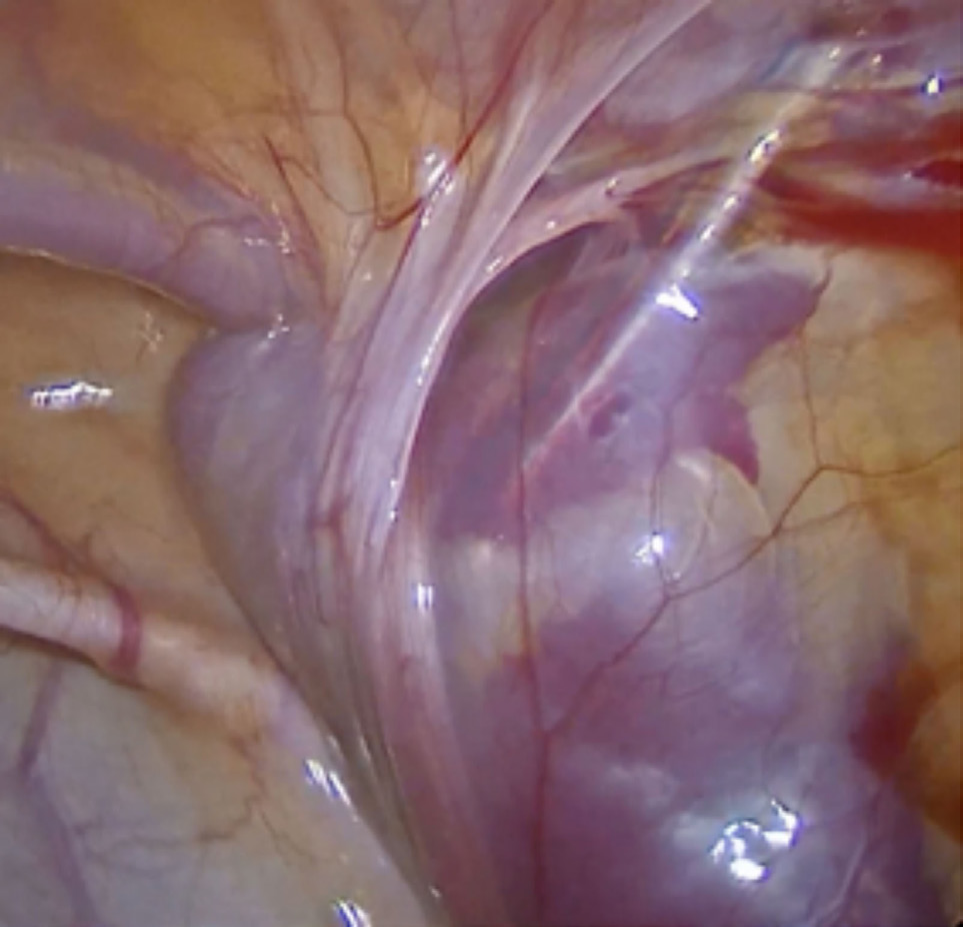
Femoral vein puncture, a perioperative complication. The procedure was interrupted, and the bleeding was controlled with external compression.

**Figure 2:**
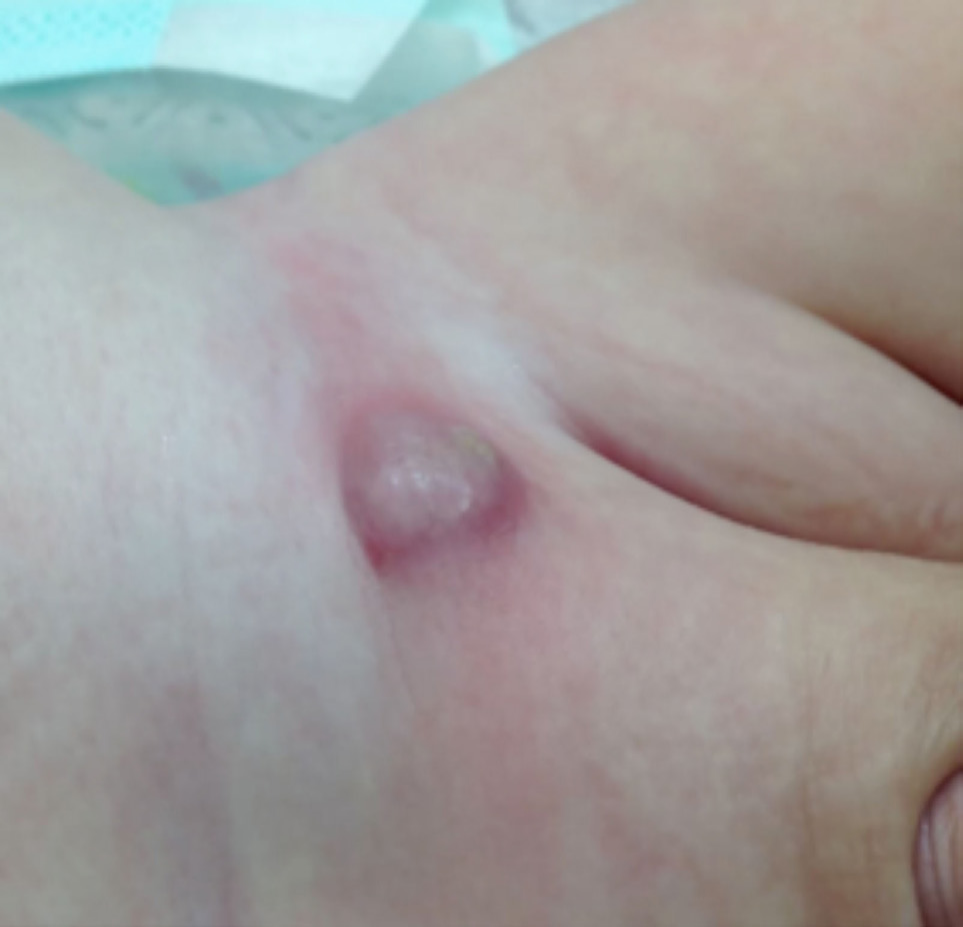
Inguinal foreign-body reaction, a postoperative complication emerging 4 weeks after surgery.

Demographic data and clinical details were gathered, including gender, age, diagnosis (hernia vs. communicating hydrocele), pre- vs. perioperative laterality match, identification of silent patent *processus vaginalis*, conversion to open repair, ipsilateral recurrence and metachronous contralateral hernia, perioperative complications reported by the surgical team such as puncture of femoral vessels (Figure 1), and postoperative complications that caused early return to the hospital, such as hematoma, wound infection, or foreign-body reactions (Figure 2).

### Operative Techniques

Both techniques were performed under general anesthesia (laryngeal mask) with the patient lying in a supine position.

#### OA Group

For open repair, we used a classic technique that divides the sac and closes the peritoneum at the level of the internal inguinal ring after opening the skin, Scarpa’s fascia, and the aponeurosis of the external oblique muscle.

#### PIRS Group

The procedures were performed under general anesthesia (laryngeal mask) with the patient lying in a supine position. The surgeon stood at the right side of the patient regardless of the affected side, and the monitor was placed at the bottom of the table. Our minimally invasive approach included the ligation of the *processus vaginalis* based on PIRS technique as described by Patkowsky et al. (11) (see Video S1 in Supplementary Material). Briefly, it included a single transumbilical incision for a 5-mm trocar (30° optics). In males, we introduced a 3-mm dissection grasper above the trocar for the optics through a stab incision across the *linea alba* to help in mobilization of the peritoneum. Insufflation pressure was between 6 and 10 mmHg depending on the age of the patient. The peritoneal cavity was inspected to confirm the diagnosis. If there was a contralateral patent *processus vaginalis* or a different type of hernia, it was repaired in the same intervention. Either a 16G Abbocath or a 18-G hypodermic needle (depending on surgeon preference), armed with a loop of Prolene^®^ 2-0 thread, was introduced through the skin at the level of the deep inguinal ring. Under laparoscopic guided vision, the needle was passed extraperitoneally between the peritoneum and the *vas deferens* and testicular vessels, leaving no peritoneal gaps, along half of the internal inguinal ring. Then the needle punched the peritoneum and entered the peritoneal cavity to push the thread through the barrel of the needle into the peritoneal cavity, forming an intraperitoneal loop. The needle was pulled out of the abdominal cavity leaving the Prolene^®^ loop inside. The needle was introduced again, through the previous skin puncture point, this time armed with an Ethibond^®^ 2-0 thread, which dissected the peritoneum of the other half of the ring, and passed through the peritoneal opening created before. The Ethibond^®^ was pushed inside the Prolene^®^ loop and the needle was taken off. From outside, the Prolene^®^ loop was pulled out of the patient’s body taking the Ethibond^®^ end with it. Both Ethibond^®^ ends were exiting the skin through the same puncture point and a perfect Ethibond^®^ cerclage was created around the internal inguinal ring leaving no peritoneal gaps. During the whole procedure, extreme care was taken not to damage the *vas deferens*, testicular, epigastric, or femoral vessels. In girls, the round ligament was included into the cerclage. The knot was tied extracorporeally and buried under the skin. Steri-strip^®^ were used over the skin puncture point at a cartoon fashion. The umbilical access was closed with absorbable suture and covered with a waterproof dressing. All patients were discharged home at the same day of surgery unless they had clinical contraindication.

### Appraisal of the Minimally Invasive Program Implementation (Learning Curves)

Global data regarding both OA and PIRS groups were analyzed and compared when appropriate. To assess the success of implementation of the minimally invasive program for repairing inguinal hernia and communicating hydrocele, the learning curves were studied in two different ways: through a department- and a surgeon-centered analysis. The intervention on each patient was always considered a single procedure independently of being unilateral and bilateral repairs.

In the department-centered analysis, the OA group was used to set the benchmarks of the department. PIRS group was divided in chronological sequential tertiles (PIRS 1st–114th; PIRS 115th–228th; PIRS 229th–341st). The following rates were calculated for each tertile: i. perioperative complications (%); ii. postoperative complications (%); iii. ipsilateral recurrence (%); iv. conversion to open repair (%); and v. males benefiting from PIRS (%). The first three rates aimed to assess either the efficacy of the technique and the expertise of the surgical team. The other two rates mainly assessed the belief of the surgical team on the benefits of the procedure and their own self-confidence in performing the technique. The tertiles were compared with each others and with the benchmark (when appropriate).

In the surgeon-centered analysis, the staff surgeons who adopted PIRS as the technique of choice were selected, in order to achieve individual sequential case series. For each surgery serial number, we calculated the rate of perioperative complications, ipsilateral recurrence, and conversion to open surgery. The results were displayed in a surgeon’s cumulative experience chart. A visual analysis was performed based on the events decline to determine the serial number of cases required to complete the learning curve.

### Data Analysis

Data analysis was performed using SPSS software version 24.0 (SPSS, Chicago, IL, USA). Chi-square test was used to compare the distribution of categorical variables between groups. Statistical significance was defined as a two-sided *p*-value < 0.05.

## Results

Six hundred seven cases matched the inclusion criteria and were included in this study (Table 1). Even though the mean ages had been similar in both groups, the rate of male gender was lower in PIRS group, whereas a higher percentage of hydrocele cases fell in OA group. This clearly suggest a case-selection bias introduced by the surgical team during their learning curve based on beliefs and confidence in the PIRS technique. In cases of unilateral hernia (or hydrocele) treated by PIRS, we could identify a contralateral silent patent *processus vaginalis* (patent *processus vaginalis* without previous diagnosis) in around one-fifth of the cases. Moreover, we identified a mismatch with preoperative laterality (when laterality was not confirmed or no patency was identified preoperatively) in 4.4% of children. Three cases of PIRS group were converted to open surgery due to technical difficulty. Reported perioperative complications (puncture of femoral vessels) were not statistically different between OA (*n* = 1; 0.4%) and PIRS groups (*n* = 8; 2.3%), yet we identified a slight tendency for higher rate in PIRS group, but regarding postoperative complications (hematoma, wound infection, and foreign-body reactions) the rates were clearly similar in both groups (*n* = 6; 2.3% and *n* = 9; 2.6%). Metachronous contralateral recurrence was significantly lower in the PIRS group (*n* = 1; 0.3% vs. *n* = 14; 5.3%), whereas PIRS ipsilateral recurrences were not different from the OA group (*n* = 5; 1.5% and *n* = 1; 0.4%).

**Table 1:** Demographic characteristics and clinical outcomes of the open approach (OA) and percutaneous internal ring suturing (PIRS) groups.

	Open group (*n* = 266 pts)	PIRS group (*n* = 341 pts)	*p*-Value
**Preoperative characteristics**			
Male gender, no. (%)	228 (86%)	216 (63%)	
Age, mean (SD), years	3.8 ± 3.5	4.2 ± 3.4	
Diagnosis, no. (%)			
Hernia	164 (62%)	311 (91%)	
Hydrocele	102 (38%)	30 (9%)	
**Perioperative results**			
Silent patent *processus vaginalis*, no. (%)	–	58 (17%)	
Mismatch with preoperative laterality, no. (%)	–	15 (4.4%)	
Conversion, no. (%)	–	3 (0.9%)	
Reported perioperative complications, no. (%)	1 (0.4%)	8 (2.3%)	0.085
**Postoperative outcomes**			
Postoperative complications, no. (%)	6 (2.3%)	9 (2.6%)	0.799
Ipsilateral recurrence, no. (%)	1 (0.4%)	5 (1.5%)	0.238
Metachronous recurrence, no. (%)	14 (5.3%)	1 (0.3%)	<0.001

*Pts, patients; no, number*.

### Department Learning Curve

**Figure 3:**
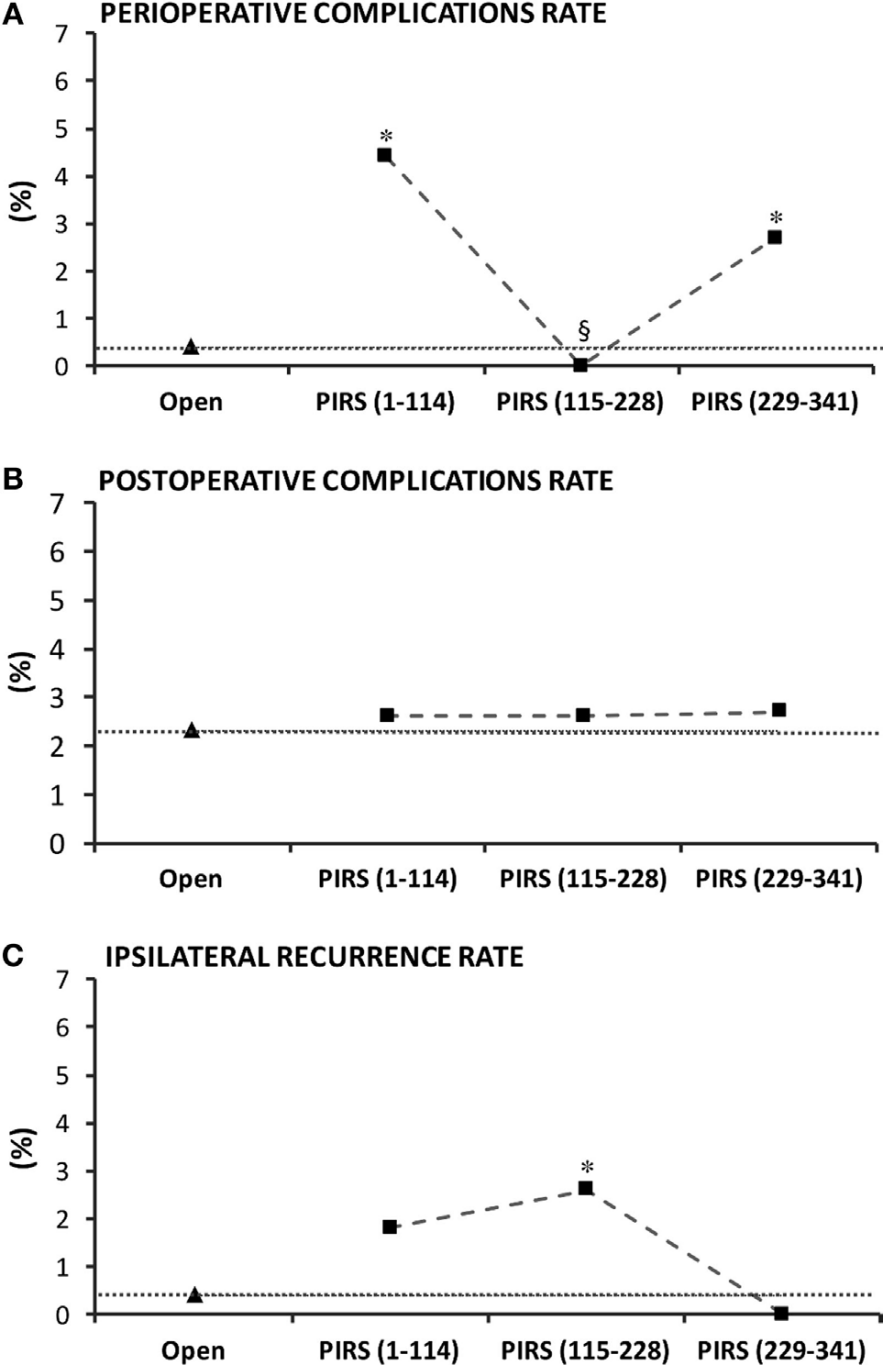
Department-centered analysis of the learning curve of percutaneous internal ring suturing (PIRS) technique considering **(A)** perioperative and **(B)** postoperative complications rates and **(C)** ipsilateral recurrence rate. *p* < 0.05 indicated significance: * vs. open; ^§^ vs. lap (0–114).

**Figure 4:**
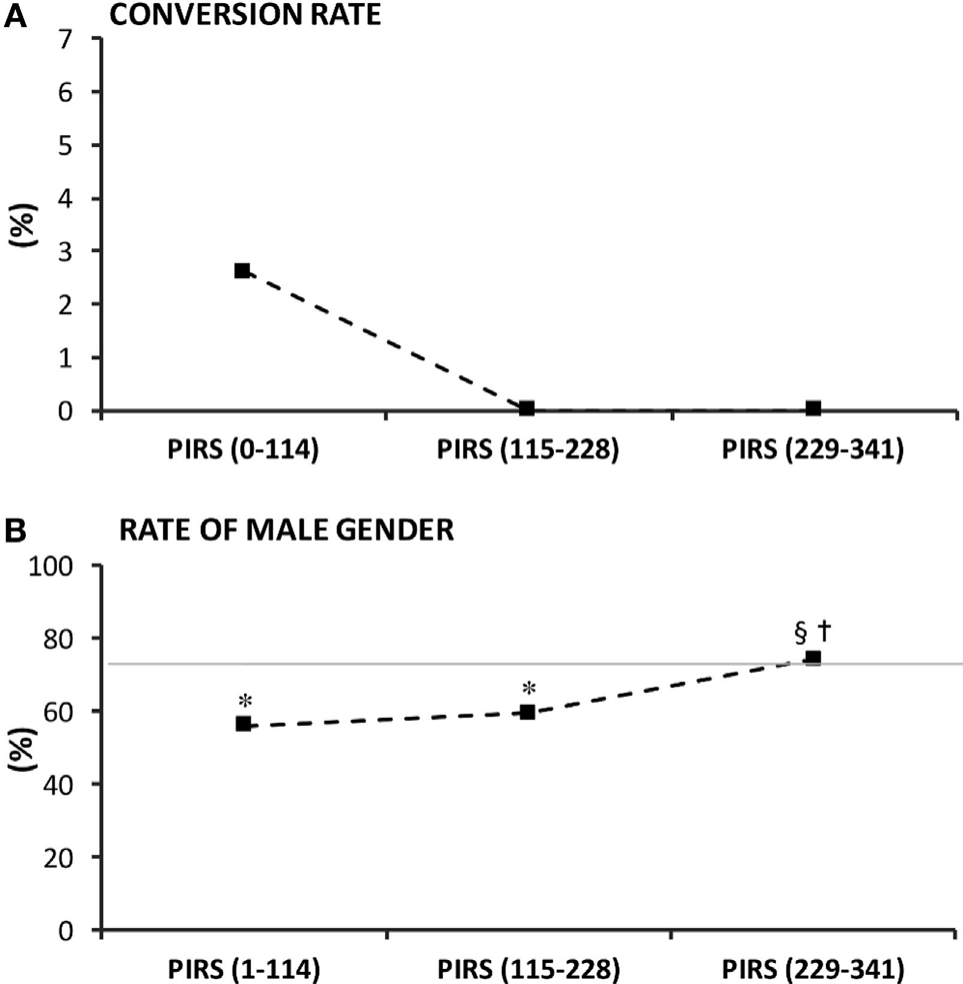
Confidence of the surgical team on the technique reflected by **(A)** conversion to open surgery rate, and **(B)** rate of male gender proposed for percutaneous internal ring suturing (PIRS). In **(B)**, the full horizontal line represents the overall rate of males among our population. *p* < 0.05 indicated significance: * vs. overall prevalence; ^§^ vs. lap (0–114); ^†^vs. lap (114–228).

In the department-centered analysis, perioperative complications rate was significantly greater than the benchmark in the first and third tertiles (Figure 3A), whereas the rate of postoperative complications was consistently identical between each tertile and the benchmark (around 2.5%) (Figure 3B). Ipsilateral recurrence rate was higher in the first two tertiles and decreased in the last tertile (Figure 3C). Conversion rate was 2.6% in the first tertile and sank to 0% from the second tertile onward (Figure 4A). Overall, the percentage of males in patients proposed for inguinal hernia and/or communicating hydrocele was 73%. Interestingly, rate of males submitted to PIRS was 56% on the first tertile and boosted along further tertiles reaching the rate of our sample at the third tertile (Figure 4B).

### Individual Learning Curve

**Figure 5:**
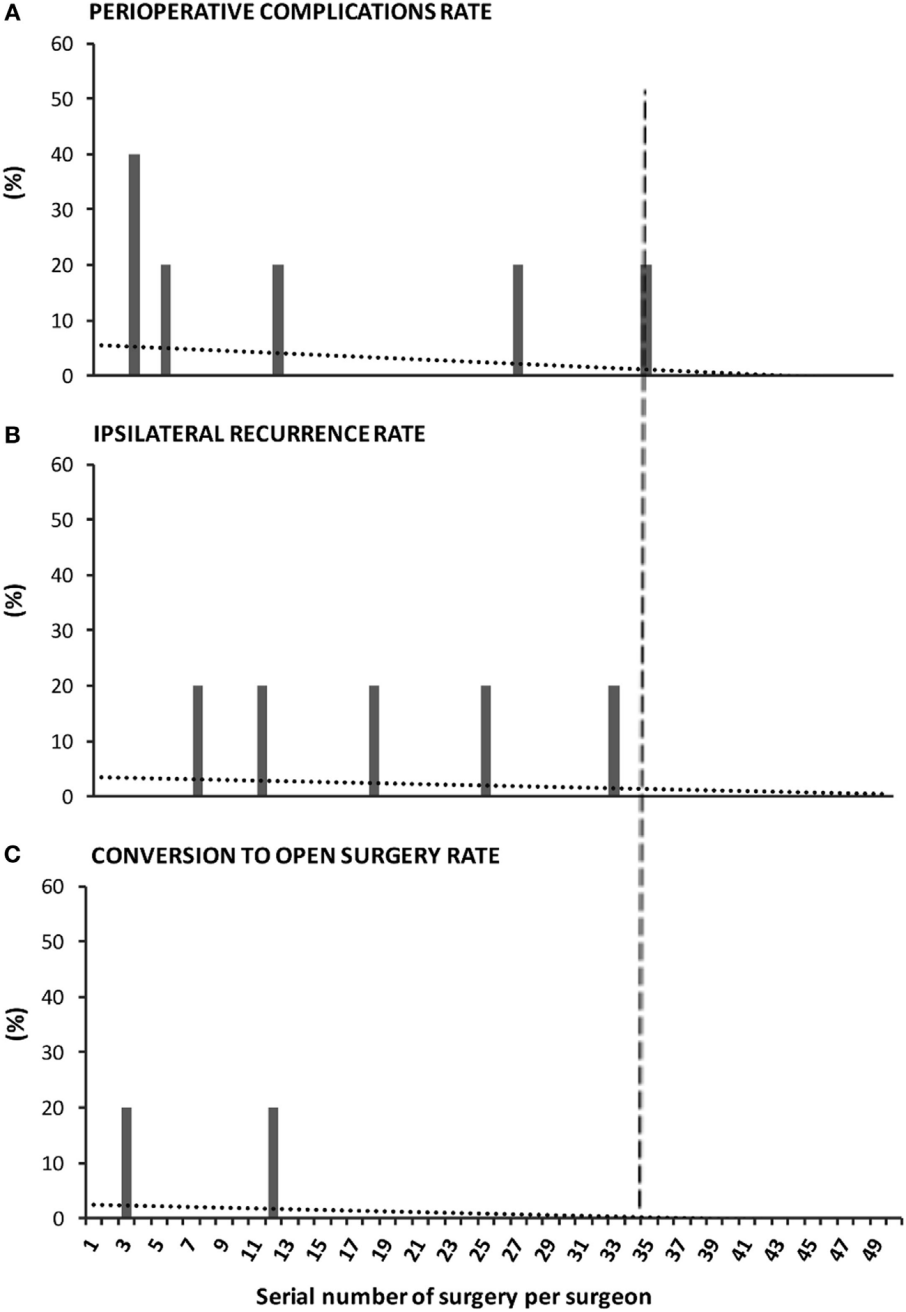
Surgeon-centered analysis of the learning curve of percutaneous internal ring suturing (PIRS) technique. The graph reflects the cumulative experience of five staff surgeons throughout their first 50 surgeries by PIRS. The performance was evaluated by the **(A)** rate of perioperative complications, **(B)** rate of ipsilateral recurrence, and **(C)** rate of conversion to open surgery. The dotted lines represent the tendency lines. The dashed vertical line crossing the *x*-axis at point 35 marks the end of the learning curve, as no events occur after the 35th case of each surgeon.

Out of six surgeons beginning the program only five adopted PIRS as the technique of choice. Each of these performed a minimum of 50 surgeries. The results chart (Figure 5) reflected the cumulative experience of the five selected surgeons along their first 50 PIRS procedures. A decline of the events was observed along the accumulated individual experience: perioperative complications reached its nadir at 35th surgery (Figure 5A). No recurrence occurred after each surgeon’s 33rd case (Figure 5B) and there were no conversions after the 12th case of each surgeon (Figure 5C).

## Discussion

This study assessed the evolution of the department and surgeon’s learning curves during a minimally invasive program to repair inguinal hernia and communicating hydrocele in children. The program was presented 5 years ago to our staff surgeons (all with different experience in minimally invasive procedures) and the proposed benchmarks were leastwise the results we had in the classical open repair. The selected minimally invasive technique was the PIRS (11) leaving no peritoneal gaps in the sac ligation at the level of the internal inguinal ring. As potential benefits, we found a better cosmesis with minimal dissection of the *vas deferens* and spermatic vessels, and the possibility to examine and fix the eventual patency of the contralateral internal inguinal ring. Meanwhile, we were aware of the increased risk of peri- and postoperative complications as well as of higher ipsilateral recurrence rate (17, 18). The drawback in operative time, inherent to every learning curves, tends to decrease along cumulative experience and was not the focus of this study.

In the department-centered learning curve analysis, five of the six staff members adhered to the program. However, there was a disparity on the implementation cadence by each of them. Therefore, we also performed a surgeon-centered learning curve analysis.

The cosmesis was excellent with our strategy to insert the optics and dissecting 3-mm instrument through the umbilicus. In fact, there were no visible scars at the end of the procedure. In the department analysis, perioperative complications and ipsilateral recurrence rates showed some undulant pattern, despite the downward trend along the tertiles. The underlying explanation might be the surgeons’ cadence disparity when starting their learning process, as each surgeon’s accumulated experience contributing to each tertile was not the same. When analyzing the surgeon-centered learning curves, we perceived a more consistent decline in perioperative complications and ipsilateral recurrence with experience. In contrast to perioperative complications, ipsilateral recurrence, and conversion rates, the postoperative complications rate was consistently similar along all tertiles (PIRS group) and the benchmark (OA group). We concluded that postoperative complications are not dependent on the learning curve, but on the technique *per se*, and most likely occur due to the type of thread, knots not deeply buried under the skin, dressing gown care, or aseptic measures. The most common postoperative complications were incisional infections and foreign-body reactions. Interestingly, most inguinal foreign-body reactions in PIRS group occurred around 2 months after the procedure and the only measure that could resolve it was the removal of the stitch, which did not determine recurrence in the majority of cases. Likely the associated inflammation promoted effective and definitive closure of the defect.

In the literature, PIRS technique had been associated with higher rates of ipsilateral recurrence and residual hydroceles (9, 17, 18) This had been attributed to several factors such as the inexperience of the surgeons, the use of absorbable suture for ligation of the patent *processus vaginalis*, the use of a single-suture, larger defects and the presence of other factors (i.e., chronic cough, constipation) (19–21). More recent publications report lower recurrence and complications rate (22, 23). Before starting the program, we discussed this issue and advanced the possibility that the ligation of the peritoneum leaving some gaps over the *vas deferens* and spermatic vessels could contribute to ipsilateral recurrence and residual hydrocele, also hypothesized by others (17). Thus, we implemented our program with recommendation to ligate the peritoneum at the level of the internal inguinal ring without leaving any gaps. For the dissection of the peritoneum over the *vas deferens* and spermatic vessels, we used the cutting tip of an Abocath 16G. This was likely the trickiest step of the procedure. Indeed, at the beginning, a complete cerclage with no gaps was not always possible to achieve, which perhaps justified the higher ipsilateral recurrence rate observed during the first tertile. Moreover, the surgical maneuvers to perform this type of ligation, in some cases, led to puncture/pierce of the femoral vessels. This was the most common perioperative complication and the main cause for conversion in the first tertile, consistently with other studies in the literature (17). In fact, this was the outcome that mostly demanded experience, once at least 35 procedures by surgeon were necessary to minimize it. To avoid this disturbing complication, some staff members used a blunt needle (hypodermic needle) which carries less risk of laceration, although it has the risk to drag or/and entrap the *vas deferens*. Meanwhile, we emphasize that ligation of the peritoneum at the level of the internal inguinal ring with no peritoneal gaps might be a determinant factor for the low ipsilateral recurrence rate and absence of residual hydrocele observed in the last tertile of patients. Incidentally, we had a successful experience fixing communicating hydroceles with this technique. It was interesting to testify that large defects in small infants, where we just ligated the peritoneum without reducing the internal ring muscular defect, evolved very well with no recurrence. This raises the necessity to ascertain unknown mechanisms led by the peritoneum in the process of anatomic–physiologic closure of the patent *processus vaginalis*.

The laparoscopic approach allows the identification of contralateral patency of the *processus vaginalis* and its repair within the same surgical intervention. This strategy is likely the leading cause for the almost disappearance of metachronous contralateral hernia as we observed a significant reduction from 5.3% (OA group) to 0.3% (PIRS group) in our series (*p* < 0.001). This outcome is intrinsic to the technique, not dependent of learning curve, as it was observed since the first cases of laparoscopic repair. It is well known that patency of the *processus vaginalis* not always manifests as a hernia or a hydrocele, and because of that, there is no consensus whether it should or not be closed (16, 24). The principal argument to leave a patent *processus vaginalis* untouched is to avoid complications (25). Interestingly, in our series, we had no complications related with the closure of the contralateral patent *processus vaginalis*. In fact, the closure of an asymptomatic patent *processus vaginalis* was technically less demanding as the patency tends to be smaller. In PIRS group, we identified a prevalence of 17% asymptomatic patent *processus vaginalis* that were fixed within the same surgical intervention, while in the OA group, metachronous hernia occurred in 5.3% of the patients, during follow-up. We speculate that almost a third of the metachronous hernias are avoidable, if a laparoscopic approach with identification and closure of an asymptomatic patent *processus vaginalis* is adopted.

Finally, a deep analysis of our data suggested that the main reasons generating some distress among surgeons for proposing a minimally invasive approach in the beginning of the program were the male gender and the youngest ages of infants. In fact, we could verify that the percentage of males with inguinal hernia or communicating hydroceles proposed for minimally invasive repair increased along the tertiles and reached the benchmark (percentage of males with inguinal hernia or communicating hydroceles in our population) only at third tertile. At the beginning of the program, surgeons selected female patients to start with as their anatomy appears more favorable (2). However, we emphasize that most recurrences, in procedures performed by less experienced surgeons, occurred in females, because in many of them there is a fold of peritoneum under the round ligament that might easily be missed during cerclage that intends to leave no peritoneal gaps. Also interesting, we realized we could use the gap between the percentage of males in our population and the percentage of males in those proposed for laparoscopic repair as an index to monitor the confidence of the surgical teams that decide to adopt a minimally invasive repair program.

In conclusion, our study demonstrates that independently of previous surgical experience in minimally invasive surgery, pediatric surgeons easily adhere to the implementation of a minimally invasive program to repair inguinal hernia and communicating hydrocele. In contrast to postoperative complications, which were technique and experience independent, there was a learning curve for perioperative complications, ipsilateral recurrence, and conversion rates that reached the nadir after each surgeon performed at least 35 cases. After this, the laparoscopic repair is a safe and effective approach, whereas the cosmesis and the virtual extinction of metachronous contralateral hernia were the major advantages. The gap between the percentage of males in those proposed for surgical repair and the percentage of males in patients operated by PIRS can be used as an index to monitor the confidence of the surgical teams that decide to adopt a similar program to repair inguinal hernia and communicating hydrocele by minimally invasive surgery.

## Ethics Statement

This study was carried out in accordance with the recommendations of the Declaration of Helsinki with written informed consent from all subjects. The protocol was approved by the scientific ethic committee from our institution.

## Author Contributions

The study conception was performed by CB, RL-P, and JC-P, data acquisition by CB, PE, AA, and JC. For interpretation of data and analysis, CB, AO, RL-P, and JC-P were involved. The manuscript was written by CB and JC-P and revised by JC, RL-P, and JC-P.

## Conflict of Interest Statement

The authors declare that the research was conducted in the absence of any commercial or financial relationships that could be construed as a potential conflict of interest.

## Supplementary Material

Video S1 Percutaneous internal ring suturing leaving no peritoneal gaps.
